# An innovative approach to reducing pain in patients with peripheral neuropathy related to HIV: A single case study

**DOI:** 10.4102/ajod.v3i1.118

**Published:** 2014-08-07

**Authors:** Natalie A. Benjamin, Jennifer Jelsma

**Affiliations:** 1 Department of Physiotherapy, School of Therapeutic Sciences, University of the Witwatersrand, South Africa; 2 Department of Health and Rehabilitation Sciences, University of Cape Town, South Africa

## Abstract

**Introduction:**

Peripheral neuropathy (PN) is the most common neurological condition seen in people living with human immunodeficiency virus (HIV) and has been estimated to be present in up to 30% of people with AIDS. Prevalence may be increasing as it appears to be related to the use of antiretroviral therapy in many cases, particularly medication containing stavudine. It is often characterised by burning and numbness in the feet. It can interfere severely with function and sufferers resort to a large number of strategies in an attempt to reduce pain.

**Case presentation:**

A 50-year-old man presented with severe PN and showed symptoms of extreme weakness and sensitivity of both lower limbs. His symptoms gradually worsened over a period of 18 months to such an extent that he was unable to walk. Medication had no effect on his pain or related symptoms. The authors tested the use of a Vibromat clinical vibrator to treat his PN symptoms. The patient was treated using the same modality for five evenly spaced (monthly) sessions.

**Outcome:**

The patient showed marked improvement in pain, numbness and pins and needles symptoms after the first session. He was able to walk a short distance with assistance. Treatment was continued and after the third session he was able to walk unaided. Symptom relief was maintained for 4 months.

**Conclusion:**

This is the first case demonstrating the use of the Vibromat in successfully treating the signs and symptoms of PN. This is important for clinicians who manage patients with HIV-related PN and pain.

## Introduction

Peripheral neuropathy (PN) is the most common neurological condition seen in people living with human immunodeficiency virus (HIV) (Kamerman, Wadley & Cherry 2012; Nicholas, Kemppainen & Canaval 2007) and has been estimated to be present in up to 30% of people with AIDS (Griswold *et al*. 2005). Prevalence was even higher (57%) in a study conducted by Wadley *et al*. (2011). Prevalence may be increasing as it appears to be related to the use of antiretroviral therapy in many cases, particularly medication containing stavudine (Oshinaike *et al*. 2012; Wadley *et al*. 2011). It is often characterised by burning and numbness in the feet (Davis *et al*. 2004). It can interfere severely with function and sufferers resort to a large number of strategies in an attempt to reduce pain (Griswold *et al*. 2005).

Limited evidence exists for the effective pharmacological management of the condition. A systematic review of pharmacological treatment of painful HIV-associated sensory neuropathy (HIV-SN) concluded that ‘[*e*]valuation of novel management strategies for painful HIV-SN is urgently needed’ (Phillips *et al*. 2010:e14433; see also Chetty *et al*. 2012). Chetty *et al*. (2012) revisited this concept and provided pharmacological guidelines based on existing literature. Kamerman and Mitchell (2011) estimate that at least 10 million HIV-positive individuals live with significant pain because of their disease. This is a rather large population that is being underserviced as a result of a lack of effective management strategies, including sufficient evidence for physiotherapy intervention.

Physiotherapy management, consisting of joint mobilisation, massage and the use of micro-current therapy, has been reported to be effective in two patients; however, the impact of therapy was only apparent after several months (Gale 2003). Nicholas *et al*. (2007) reported that one of the coping strategies of patients with PN was rubbing the feet with cream, which had a 6.3/10 reported effectiveness (Nicholas *et al*. 2007). Both Gale (2003) and Nicholas *et al*. (2007) report on the effectiveness of intervention which included tactile stimulation, albeit not in experimental trials. The current authors hypothesised that the use of vibration, which provides a strong sensory stimulus, might reduce the pain and reduced sensation common in patients with PN.

The use of vibration to reduce pain has been suggested in dental patients and rests on a basic application of the gate control theory of pain (Shahidi Bonjar 2011). This theory proposes that stimulation of small C fibres activates excitatory systems that excite pain output cells. These cells’ activity is controlled by the balance of proprioceptive, large fibre, A beta mediated inhibitions (Dickenson 2002), which implies that proprioceptive input can assist in preventing the transmission of noxious stimuli to the central nervous system. A similar case study was conducted by Hong (2011), where whole-body vibration was used to manage diabetes-related neuropathic pain. Following an eight-week intervention of whole-body vibration, the patient’s pain level, balance and gait patterns were significantly improved.

As this is a case study and the results were somewhat unexpected, we had not anticipated submitting for publication and ethical approval could not be granted retrospectively. However, consent to publish this article was obtained from the subject, provided that no identifying details were disclosed.

## Medical history

Mr X was a 50-year-old male who was first seen by the authors on 14 November 2012. He had been diagnosed with pulmonary tuberculosis (TB) and HIV with severe PN in May 2011, 5.5 months previously. He was commenced on HAART – D4T, 3TC and EFV on 05 July 2011. He was admitted to Mahatma Ghandi Memorial Hospital on 12 November 2012 for extreme weakness and sensitivity of both lower limbs. The patient reported that he had not been walking for over a year and was dependent on a wheelchair for mobility as well as caregivers for transfers. He was referred to physiotherapy by the attending doctor to assist with mobilising the patient out of bed and pain management. He was receiving medication as listed in Table 1.

**TABLE 1:** In-hospital medication regime.

Medication	Dosage	Indication
Tegretol	20 mg Nocté	Nerve pain
Panado	500 mg TDS	Pain
Tenofovir (TDF)	300 mg D	Anti-retroviral therapy
Efavirenz (EFV)	60 mg D	Anti-retroviral therapy
Folate	3 g D	Fatigue
Amitriptyline HCL	25 mg Nocté	Depression, sedative effect to aid sleeping and pain management

HCL, hydrochloride.

He had undergone lumbar spine X-rays to exclude TB spine and his vitamin B levels were within the normal range.

### Social history and environmental factors

Mr X was married and unemployed and received a social grant. He felt very depressed and demotivated because of his limitations and the severe pain.

He lived in a five-room house, with an outside toilet which he was unable to access because of his disability. He had electricity but no running water in his home.

### Subjective examination

Mr X arrived at the Physiotherapy Department in a wheelchair. He was wearing his slippers with doubled up socks as he found it too painful to touch the normal lining of the slippers. He was unable to transfer independently from the chair to the examination plinth and required the assistance of two helpers.

He reported severe pain in the lower limbs and that he fatigued easily. He required assistance with grooming but was independent with regard to other self-care activities. He was unable to sleep because of the pain and was unable to cover his legs with a sheet or blanket for warmth because of the hypersensitivity of his feet. His desired outcome was that he would be pain free so that he could walk again and regain his independence, specifically in terms of getting to the toilet without assistance and attending church services.

### Objective examination

He had intact biceps and triceps reflexes in the upper limb. It was not possible to test the knee and ankle jerks as a result of the presence of severe pain. He exhibited severe hypersensitivity and allodynia within the L4–S1 dermatomes. He was unable to tolerate any form of tactile stimulation, light touch, pin-prick or temperature testing and found even the touch of cotton wool unbearable. He also reported a sudden shooting pain on unexpected touch or movement. He rated his pain as 10/10 on the pain visual analogue scale (VAS).

He was generally weak (Grade 3–4 muscle strength on the Oxford Scale of muscle strength [Porter 2013]) which was likely a result of disuse of his arms and lower limbs. He had full range of movement in the upper limbs and at the hip and knee. His feet were discoloured and he had dry, scaling skin on his feet, evidence that he had not taken weight through his feet for a while. He had hammer toes which indicated paralysis of the intrinsic muscles of the foot. He was unable to do any active dorsi-flexion and could only plantar flex up to 10° as a result of pain and a degree of swelling around his ankles. The muscle strength of his ankle muscles was Grade 2 on the Oxford Scale, which by definition meant that he was unable to move his ankles through full range against gravity.

He had been receiving physiotherapy treatment, which consisted of light tactile stimulation (using cotton wool), for the previous two days in an attempt to desensitise the hyperalgesic areas. He had also received treatment to strengthen his upper limbs and to teach him how to manage his wheelchair.

### Intervention

A Vibromat clinical vibrator was utilised for intervention. This consists of a vibrating pad of approximately 10 cm x 12 cm, driven by a small motor. It is standard equipment in most physiotherapy departments as it is generally used to assist in dislodging chest secretions by vibrating over the chest wall.

#### Treatment session 1: 14 November 2012

We treated Mr X supported in supine. We covered the pad with a sterile glove and we requested Mr X to touch the Vibromat, which was set at a low frequency, in order to get used to the sensation. We started vibrating proximal and lateral to the hyperaesthetic area by placing the Vibromat next to his calf and asked him to move his calf against the pad. He was thus in control of the amount of stimulation he received. He found it painful and was hesitant to push against the pad at first. However, within a very short space of time he was able to push quite hard and was then willing to move his leg so that the pad approached his ankle and finally his foot. In the beginning he could tolerate only light touch but within approximately 60 seconds he was able to apply quite firm pressure against the pad with his ankle and ultimately the sole of his foot. The treatment lasted for approximately 5 minutes and was then repeated on the other leg.

He immediately reported a decrease in pain and he was also able to move his ankles more freely and with minimal pain. We then assisted him into standing, and for the first time in 6 or more months, he was able to walk with minimal support. He walked the length of the ward and his response was ‘Thank God, thank God!’

The response was of such magnitude and so immediate we were concerned that there might be a severe exacerbation in pain and we counselled him in this regard and warned him against doing too much.

#### Treatment session 2: 15 November 2012

The patient reported no increase in pain; on the contrary, it was much improved to 7/10 on the VAS, but had increased since the cessation of treatment the previous day. His ward mates reported that he had been walking with the assistance of other patients. The swelling in his feet had subsided completely. He then applied considerably more pressure, paying attention to the area at the ball of his left foot, which was more painful than his right. He reported that the pain had decreased to 5/10 on the VAS. He reported that he had had pins and needles prior to treatment but this subsided completely after the vibration. Post-treatment he walked with more ease, at a faster pace and with minimal assistance and was able to walk to the bathroom on the ward.

#### Treatment session 3: 18 November 2012

Improvement continued and vibration treatment was given as before. He reported a 4–5/10 pain intensity on the VAS. He was discharged from hospital with medication (see Table 2) and was given mobility and strengthening exercises to continue at home.

**TABLE 2:** Medication issued on discharge from hospital.

Medication	Dosage	Indication
Pyridoxine	25 mg D	Vitamin B6 supplement
Tenofovir (TDF)	300 mg D	Anti-retroviral therapy
Efavirenz (EFV)	600 mg D	Anti-retroviral therapy
Lamivudine (3TC)	300 mg D	Anti-retroviral therapy
Amitriptyline HCL	25 mg Nocté	Depression and pain management

HCL, hydrochloride.

#### Treatment session 4: 11 December 2012

Mr X arrived late for his appointment with the authors and was given regular physiotherapy without vibration, which included upper limb strengthening exercises, cycling for 10 minutes and stair climbing. He coped well and was issued with elbow crutches to aid his balance.

#### Treatment session 5: 8 January 2013

Mr X walked into the Physiotherapy Department with his crutches as he no longer felt the need to use his wheelchair and opted to rest if he was tired. On examination the active range of movement in his ankles had improved 10° in all directions (dorsi- and plantar-flexion and inversion and eversion) from 7 weeks before. The condition of his skin had also improved. The patient was able to climb the stairs with no walking aid. Therapy was continued as before, including the vibration. He was issued with a hand-held commercial massager for use at home. He left the Physiotherapy Department with his crutches under his left arm and smiled, saying: ‘I might just need them if I get too tired’.

#### Outpatient management

Mr X continues to receive vibration therapy, once a month, on an outpatient basis. He reports that he has maintained his level of mobility and independence. His quality of life in general has improved tremendously and he attributes this positive change directly to the physiotherapy intervention that he has received.

## Conclusion

A randomised control trial of vibration in patients with HIV and PN is being planned and it is obviously premature to present this short report based on a single case study. However, the results of the intervention were so immediate and resulted in such a large functional change that we decided to share these, in the hope that those who suffer from this severe disabling condition might benefit from a similar intervention in the interim. The findings are also complemented by the case study reported by Hong (2011).

Mr X reported:

‘Physiotherapy made a big, big difference in my life and has been the most amazing experience. I was able to enjoy Christmas with my family because my appetite is back and I can enjoy the food. I can sleep with my legs covered with a sheet and blanket. Even my wife is happy because I can now satisfy her sexually again. I only have minor pains and still get tired.’

The qualitative results of this case study pave the way for more in-depth quantitative research to investigate adequately the mechanisms and effects of local vibration therapy.

## References

[CIT0001] Chetty S., Baalbergen E., Bhigjee A., Kamerman P.R., Ouma J., Raath R. et al, 2012, ‘Clinical practice guidelines for management of neuropathic pain: Expert panel recommendations for South Africa’, *South African Medical Journal* 102, 312–325. 22554341 10.7196/samj.5472

[CIT0002] Davis L., Evans S., Fishman B., Haley A. & Spielman L.A, 2004, ‘Predictors of attrition in HIV-positive subjects with peripheral neuropathic pain’, *AIDS Care* 16(3), 395–402. http://dx.doi.org/10.1080/09540120410001665394 15203432 10.1080/09540120410001665394

[CIT0003] Dickenson A.H, 2002, ‘Gate control theory of pain stands the test of time’, *British Journal of Anaesthesia* 6, 755–757. http://dx.doi.org/10.1093/bja/88.6.755 10.1093/bja/88.6.755 12173188

[CIT0004] Gale J, 2003, ‘Physiotherapy intervention in two people with HIV or AIDS-related peripheral neuropathy’, *Physiotherapy Research International* 8(4), 200–209. http://dx.doi.org/10.1002/pri.290 14730724 10.1002/pri.290

[CIT0005] Griswold G.A., Evans S., Spielman L. & Fishman B, 2005, ‘Coping strategies of HIV patients with peripheral neuropathy’, *AIDS Care* 17(6), 711–720. http://dx.doi.org/10.1080/09540120412331336715 16036257 10.1080/09540120412331336715

[CIT0006] Hong J, 2011, ‘Whole body vibration therapy for diabetic peripheral neuropathic pain: A case report’, *Health Science Journal* 1(5), 66–71.

[CIT0007] Kamerman P.R. & Mitchell D, 2011, ‘Current perspectives on HIV-related pain and its management: Insights from sub-Saharan Africa’, *Future Medicine* 1(16), 587–596. 10.2217/pmt.11.65 24645769

[CIT0008] Kamerman P.R., Wadley A.L. & Cherry C.L, 2012, ‘HIV-associated sensory neuropathy: Risk factors and genetics’, *Current Pain and Headache Reports* 16, 226–236. http://dx.doi.org/10.1007/s11916-012-0257-z 22367397 10.1007/s11916-012-0257-z

[CIT0009] Nicholas P., Kemppainen J. & Canaval G, 2007, ‘Symptom management and self-care for peripheral neuropathy in HIV/AIDS’, *AIDS Care* 19(2), 179–189. http://dx.doi.org/10.1080/09540120600971083 17364396 10.1080/09540120600971083

[CIT0010] Oshinaike O., Akinbami A., Ojo O., Ogbera A., Okubadejo N. & Ojini F, 2012, ‘Influence of age and neurotoxic HAART use on frequency of HIV sensory neuropathy’, *AIDS Research and Treatment* 2012, 961510 http://dx.doi.org/10.1155/2012/961510 22570772 10.1155/2012/961510 PMC3337556

[CIT0011] Phillips T.J.C., Cherry C.L., Cox S., Marshall S.J. & Rice A.S.C, 2010, ‘Pharmacological treatment of painful HIV-associated sensory neuropathy: A systematic review and meta-analysis of randomised controlled trials’, *PLoS ONE, Public Library of Science* 5(12), e14433. 10.1371/journal.pone.0014433 PMC3010990 21203440

[CIT0012] Porter S, 2013, *Tidy’s Physiotherapy*, Churchill Livingstone, Edinburgh.

[CIT0013] Shahidi Bonjar A.H, 2011, ‘Syringe micro vibrator (SMV), a new device being introduced in dentistry to alleviate pain and anxiety of intraoral injections, and a comparative study with a similar device’, *Annals of Surgical Innovation and Research* 5(1), 1 http://dx.doi.org/10.1186/1750-1164-5-1 21211061 10.1186/1750-1164-5-1 PMC3025000

[CIT0014] Wadley A.L., Cherry C.L., Price P. & Kamerman P.R, 2011, ‘HIV neuropathy risk factors and symptom characterization in stavudine-exposed South Africans’, *Journal of Pain and Symptom Management* 41(4), 700–706. http://dx.doi.org/10.1016/j.jpainsymman.2010.07.006 21145196 10.1016/j.jpainsymman.2010.07.006

